# Granulocyte-macrophage colony-stimulating factor as an immune-based therapy in HIV infection

**DOI:** 10.1186/1476-8518-3-3

**Published:** 2005-05-18

**Authors:** Pierre Antoine Brown, Jonathan B Angel

**Affiliations:** 1 Department of Medicine, University of Ottawa, 501 Smyth, Box 210, Ottawa, Canada, K1H 8L6; 2 Division of Infectious Diseases, Ottawa Hospital – General Campus, 501 Smyth, Room G-12, Ottawa, Canada, K1H 8L6

## Abstract

The HIV/AIDS epidemic continues to spread despite more than 20 years of significant research and major advances in its treatment. The introduction of highly active antiretroviral therapy in recent years has significantly improved disease treatment with a dramatic impact in HIV/AIDS associated morbidity and mortality in countries which have access to this therapy. Despite these advances, such therapies are imperfect and other therapeutic modalities, including immune-based therapies, are being actively sought. Potential benefits of immune-based therapies include: 1) the improvement of HIV-specific immunity to enhance control of viral replication, 2) the improvement of other aspects of host immunity in order to prevent or delay the development of opportunistic infections and 3) the potential to purge virus from cellular reservoirs which are sustained despite the effects of potent antiretroviral therapy. Granulocyte-macrophage colony-stimulating factor (GM-CSF) has been studied as one of these immune-based therapies. Several randomized, controlled trials have demonstrated benefits of using GM-CSF as an adjunct to conventional anti-retroviral therapy, although such benefits have not been universally observed. Individual studies have shown that GM-CSF increases CD4+ T cells counts and may be associated with decreased plasma HIV RNA levels. There is limited evidence that GM-CSF may help prevent the emergence of antiretroviral drug resistant viruses and that it may decrease the risk of infection in advanced HIV disease. Despite its high costs and the need to be administered subcutaneously, encouraging results continue to emerge from further studies, suggesting that GM-CSF has the potential to become an effective agent in the treatment of HIV infection.

## Review

### Introduction

More than 20 years after its discovery, and despite extensive research in the field, HIV-1 infection remains one of the most important public health problems in the world. The HIV/AIDS epidemic continues to spread and an increasing number of people continue to live with HIV/AIDS and die from it. The advent of highly active antiretroviral therapy (HAART) marked a cornerstone in HIV/AIDS treatment that drastically changed the prognosis of HIV infection, by its ability to induce sustained suppression of viral replication [1-4]. Yet HIV infection remains, to this day, incurable. Even with multiple available therapeutic options, failure of therapy, manifested by a rebound in plasma viral load accompanied by further decline in CD4+ T cell counts, remains frequent, leaving limited available options for the treatment of individuals experiencing such failures. The persistence of HIV infection in the face of HAART is due to its limited effect on the persistent cellular reservoir(s) of replication-competent virus. T cells and macrophages have been implicated as such reservoirs [5-7]. This discovery prompted research in the field of immune-based therapy, in the hopes of enhancing or restoring cell mediated immune responses to HIV, or even purging latent viral reservoirs. A number of different approaches have been and are being studied, including several cytokines and therapeutic vaccines that are at various stages of evaluation in human trials [8-10]. Only a limited numbers of these have however been evaluated in controlled clinical trials and only interleukin-2 (IL-2), Remune™ and GM-CSF have been the subject of phase III studies, with clinical events as the primary outcomes [11-14]. Initially used in the treatment of leukopenia in HIV-1 infection, GM-CSF has also been used in clinical trials as an adjunct to HAART in which some of the results appear promising [12,15-18]. In this review, results from published randomized controlled trials that have evaluated the potential role for GM-CSF in the management of patients with HIV infection will be summarized (see Table in Additional file: 1).

### Pre clinical and early clinical studies and the rationale for GM-CSF as an adjunctive treatment in HIV infection

GM-CSF is a pleiotropic growth factor that enhances the number and function of various cells from both the myeloid and lymphoid lineages, including neutrophils, monocytes and lymphocytes [19]. It is one of the many cytokines profoundly affected by HIV infection with its production being significantly reduced [20,21]. This has been one of several rationales for its use in HIV-infection. First, replacement therapy is seen as a way of enhancing the bone marrow's production of cells important in cell-mediated immunity, including CD4+ lymphocytes. Second, GM-CSF has also been shown *in vitro *to enhance the activity of the antiretroviral agent zidovudine (AZT) in macrophages [22,23] and thus may be an approach to enhance clearance of viral reservoir when used in combination with HAART. Third, GM-CSF also has an effect on monocyte-derived macrophages. Maturation of monocytes into macrophages is usually accompanied by an increase in the expression of CCR5, the co-receptor for the M-tropic HIV strains, a finding that seems to explain the observation that HIV entry is more efficient in macrophages than in monocytes [24]. *In vitro*, the presence of GM-CSF suppresses the expression of CXCR4 mRNA and CCR5 mRNA by monocytes differentiating in macrophages, resulting in macrophages that are relatively resistant to M-tropic HIV infection [25].

In addition to *in vitro *studies that have suggested that GM-CSF enhances the action of anti-retroviral drugs (ARVD) in macrophages [22,23], data supports the idea that GM-CSF can also lower the frequency of ARVD-resistant HIV-1 mutants *in vivo*. There appears to be lower frequency of resistant-mutations among subjects on zidovudine and GM-CSF, as part of their anti-retroviral regimens, versus those on AZT alone [16]. This finding is of potential significance, as the management of drug resistant strains of HIV remains a major issue. However, which specific mutations were observed at what frequency was not reported. This has an impact on the importance of this finding, as not all mutations have the same clinical significance. As well, whether these observations with AZT occur with other ARVD and how relevant this is given the current management of HIV-infected individuals remains to be established. Although it is used effectively in patients with neutropenia, typically caused by medication or bone marrow dysfunction [26-28], the positive effect of GM-CSF on CD4+ lymphocyte count in HIV had not been studied or well documented in early observational studies [29-31]. Following these *in vitro *findings and early *in vivo *studies, several randomized controlled trials were developed that studied the effect of GM-CSF as a treatment for HIV-1 infected individuals.

### Impact of GM-CSF use in HIV infected individuals

#### Effect of GM-CSF on plasma HIV RNA levels

Few randomized controlled trials of GM-CSF have shown a clear, significant reduction in HIV replication. The first randomized controlled trial on the use of GM-CSF in non-neutropenic HIV-1 infected subjects, published in 1999, did not show any significant effect of GM-CSF on plasma HIV RNA levels [15]. This trial enrolled 20 patients, ten in the placebo group and ten in the treatment group. Subjects had similar baseline characteristics; the mean HIV RNA load was 3.95 log_10 _copies/ml in the placebo group compared with 4.21 log_10 _in the treatment group (p = 0.29) and the mean CD4+ T cells count in the placebo group was 243 cells/mm^3 ^compared with 178 cells/mm^3 ^in the GM-CSF group. All subjects were on stable antiretroviral therapy, including either indinavir or ritonavir, for a mean period of 5.0 months in the placebo group and 4.8 months in the treatment group. They received either 250 μg of GM-CSF or placebo subcutaneously 3 times per week for a total of eight weeks. All subjects were followed closely every two weeks during the study and twice at week 3 and week 5 after the study ended. During the study and at both follow up time points, the viral load remained within 0.5 log_10 _copies/ml of the baseline values for both groups.

Despite no overall changes in the mean HIV RNA load between groups, more subjects in the GM-CSF group than in the control group had HIV RNA values decreased by >0.5 log_10 _from baseline (50% vs. 10%). Since this size of a viral load decrease has been associated with clinical benefits, this study suggests that GM-CSF may have a beneficial effect in a subset of individuals.

In the largest double blind, randomized controlled trial on GM-CSF use in HIV infected individuals published to date, 309 subjects stratified according to viral load (≤ 30000 copies/ml vs. > 30000 copies/ml) received either 250 μg of GM-CSF or placebo three times per week for 24 weeks [12]. In total, 70% of subjects completed the full 24-week period. In the treatment and control arm respectively, 89 and 90% were males, 80% and 79% of subjects were on at least 3 antiretroviral agents, and 82% in both groups previously had one or more opportunistic infection. The mean CD4+ T cell count was 49.8 cells/mm^3 ^in the control group and 50.8 cells/mm^3 ^in the GM-CSF group. The majority of subjects entered the study with a viral load over 30000 copies/ml (62% for the placebo arm and 63% for the GM-CSF arm). There was no significant decrease in HIV-RNA in the combined strata in either the placebo or treatment group. However, GM-CSF had a positive influence on other viral parameters. GM-CSF use delayed virologic failure in those patients with plasma HIV RNA levels less than 400 copies/ml before initiation of GM-CSF therapy. At 6 months, 24 out of 29 (83%) of subjects on GM-CSF maintained viral loads below the limit of detection compared to 15 out of 28 (54%) of those on placebo (p = 0.02). This, in turn, reduced the need for antiretroviral regimen change. In this trial, ARVD regimen changes were allowed, which could have obscured a preferential decrease in viral load by GM-CSF. As such, there were fewer changes in ARVD regimens in the GM-CSF group (19%) than in the placebo group (38%) for the lower viral load stratum (p = 0.03). In the higher stratum, no significant difference in treatment change was observed (62% placebo versus 62% GM-CSF; p = 0.68). Again, this supports the idea that low-dose GM-CSF may have the potential to limit HIV replication and prevent or delay the development of drug resistant viruses, as described earlier in *in vitro *and *in vivo *studies.

In a Brazilian study, 105 individuals with AIDS were enrolled in a placebo-controlled, double-blind randomized control trial to receive AZT along with GM-CSF (125 μg) or placebo twice weekly for 6 months [16]. Subjects were required to have an AIDS defining diagnosis based on 1993 Center for Disease Control and Prevention criteria within the last three months or a CD4+ cell count <300 cells/mm^3^. Patients were excluded if they had an active AIDS defining diagnosis at the time of randomization or if they had been exposed to zidovudine for >6 months prior to study entry. The mean HIV RNA plasma levels at baseline were 93000 copies/ml in the placebo group and 155000 copies/ml in the GM-CSF group (p = 0.21). All the subjects received AZT, and 65% and 68% of subjects in the placebo and GM-CSF group respectively were also on a second agent, either ddI, ddC, 3TC or Saquinavir. Prior opportunistic infection rates were 58% in the placebo group and 70% in the treatment group (p = 0.14). This study did show a statistically significant effect of GM-CSF on viral loads. Mean HIV RNA levels declined in the GM-CSF group throughout the 6 months of the study. Over this period, the change was -0.07 log_10 _copies/ml in the control group as opposed to -0.60 log_10_copies/ml in the treatment group (95% CI -0.94-0.12; p = 0.02). As well, there was a greater number of subjects in the GM-CSF group with a decrease of 1 log_10 _or greater in viral load (20/52; 38%) compared with the placebo group (9/53; 17%) (p = 0.02). The reason why a decrease in viral load was observed in this study and not in other trials is unclear. It was the only trial with a smaller dose of GM-CSF (125 μg twice weekly vs. 250 μg thrice weekly for most other trials) and all patients were receiving AZT, both of which might have played a role in this difference.

More recent clinical data on the use of GM-CSF in combination with HAART continues to show some effect of GM-CSF on viral load [17]. These data stem from a randomized controlled trial in which 116 subjects were required to remained virologically stable (within a difference of 0.7 log_10 _copies/ml) for at least 7 days prior to entry and where no HAART regimen change was allowed during the 16 weeks period of the trial. Subjects were divided in 2 groups, depending if their CD4+ T count was below or above 200 cells/mm^3 ^at baseline and then randomized to either 250 ug of GM-CSF or placebo three times per week for 16 weeks. All patients subsequently received a 32-week course of open label GM-CSF. Baseline characteristics were similar in both groups. At baseline, in the ≥ 200 and <200 CD4+ cells/mm^3 ^strata, median plasma RNA levels were 3.81 log_10 _and 4.46 log_10 _copies/ml, with no difference between control and treatment groups. After the 16 weeks of double-blinded treatment, the change in HIV RNA levels was +0.048 log_10 _copies/ml in the GM-CSF group compared with -0.103 log_10 _copies/ml (p = 0.036) in the placebo group, both strata combined. However, when the two strata (≥ 200 and <200 CD4+ cells/mm^3^) were studied individually, the changes in mean viral loads were not significant. Thus, in this trial, subjects in the GM-CSF group, irrespective of their initial CD4+ count, tended to have a modest increase in HIV RNA levels at the end of the 16-week randomized period. Although the modest increase in viral load was significant, it was not associated with a decrease in CD4 counts or an increase in clinical events, as is discussed later.

Finally, a Swiss study evaluated the use of GM-CSF 300 μg three times a week for the first four weeks of a 12-week HAART interruption period [18]. This small study randomized 33 subjects who had previously been stable on HAART for at least six months, with viral load below 50 copies/ml and CD4+ T cell counts >400 cells/mm^3^. In both groups the viral load peaked at 6 weeks and trended down afterwards. In the GM-CSF group, the maximum viral load reached a mean of 4.97 log_10 _compared with 5.54 log_10 _in the scheduled treatment interruption-only (STI-only) group (p = 0.03). Over a period of twelve weeks, the mean area under the curve for viral loads were 47.77 log_10 _in the GM-CSF group and 51.88 log_10 _in the STI-only group (p = 0.07) This suggests not only that there is no deleterious effect of GM-CSF on plasma HIV RNA levels but that GM-CSF may help control the viral load in patients who need to stop HAART for a short period.

Overall, the evidence regarding the effect of GM-CSF on plasma HIV-1 RNA levels is somewhat conflicting. Four of the five trials reviewed show either a significant decline or no statistical changes in the viral load. The explanation for the observed increase in viral load in the GM-CSF group in the trial by Jacobson *et al. *is not clear. This study was somewhat unique in that it included only patients with uncontrolled viral replication. It appears likely that the impact of GM-CSF on viral load is dependant upon the setting in which GM-CSF is used. Furthermore, GM-CSF may selectively enhance the antiviral activity of specific antiretroviral agents (e.g. AZT). Also, as may be becoming apparent with other immune-based therapies, the greatest effect of GM-CSF may be observed in situations where there is the greatest degree of virologic suppression and associated immunologic recovery.

#### Effect of GM-CSF on CD4+ T cells

The initial randomized control trial of GM-CSF use in non-leukopenic HIV infected individuals, referred to previously, reported other important findings. CD4+ T cell counts reached higher levels in the treatment group, but these results did not reach statistical significance [15]. The mean maximal increase in the treatment group was 129.6 ± 149.9 cells/mm^3 ^and 57 ± 58.9 cells/mm^3 ^in the control group (p = 0.02). A significant majority (70%) of subjects treated with GM-CSF demonstrated an increase of >30% of their CD4+ T cell counts over baseline at any given time versus a minority (30%) in the placebo group (p = 0.07). When those patients with baseline CD4+ T cell counts of <50 cells/mm^3 ^were excluded from the analysis, in order to ensure an increase of >30% was not due to daily variability, 6 of 7 patients in the GM-CSF group and 1 of 8 patients in the placebo group had a CD4+ T cell increase of >30% (p = 0.01). This may have a clinical impact as a >30% increase of the CD4+ T cell count in light of a stable viral load has been associated with a relative risk reduction of disease progression in a previous study [32].

An earlier randomized controlled study looking into the effect of GM-CSF on leukopenia in HIV-infected individuals also demonstrated an increase in CD4+ T cell counts. After 12 weeks of therapy (300 μg GM-CSF daily for 1 week then 150 μg twice-a-week for 11 weeks), absolute CD4+ T cell counts rose by 53% compared to baseline (p < 0.001) and was statistically different than that observed in the control group (p < 0.001) [26].

Other trials observed a trend towards modest, non-significant, increases in the absolute CD4+ counts. In the study by Brites *et al*, the authors reported a modest increase in the CD4+ T cell count in both groups at six months [16]. In the GM-CSF group, there was a small, non-significant increase in the CD4+ T cell count of 35 cells/mm^3 ^compared with 12 cells/mm^3 ^in placebo group (p = 0.42). As with the study by Skowron *et al*, they also observed a significant difference in the number of subjects who had a ≥ 30% increase of the CD4+ T cell count. Only 59% of subjects in the placebo group achieved this increase as opposed to 80% in the GM-CSF group (p = 0.03).

In the other, more recent trial, from Jacobson *et al*., the authors reported a change in the CD4+ T cell count of +29 cells/mm^3 ^in the GM-CSF vs. -8 cells/mm^3 ^in the placebo group for the stratum of subjects with >200 CD4+ T cells/mm^3 ^at baseline (p = .20) [17]. They observed a similar trend in the <200 CD4+ T cells/mm^3 ^stratum, with +5 cells/mm^3 ^in the GM-CSF group at 16 weeks vs. -5 cells/mm^3 ^in the placebo group but this did not reach statistical significance (p = 0.22).

Fairly convincing evidence of a significant increase in absolute CD4+ leukocyte count following treatment with GM-CSF comes from the phase III randomized control trial by Angel and colleagues, described earlier [12]. The baseline CD4+ T counts were 49.8 × 10^6 ^cells/L for the placebo group and 50.8 × 10^6 ^cells/L for the treatment group. At 12 months the mean CD4+ T cell count was 102 ± 15 × 10^6 ^cells/L in the placebo group vs. 152 ± 18 × 10^6 ^cells/L in the treatment group. This was also reflected by statistically significantly greater increases in the CD4+ T cell count at 1, 3 and 6 months in the GM-CSF group.

It might be speculated that, since this was the largest (n = 307) and one of the longest (24 weeks) randomized control trial of GM-CSF use in HIV infected individuals, it maybe the only study with enough power to demonstrate statistical significance. As other randomized control trials show trends towards higher CD4+ T cell counts in the GM-CSF group, and statistically significant difference in various sub-analysis, it is possible that an appropriately conducted meta-analysis would clarify the impact of GM-CSF on CD4+ T cell counts.

The recent study by the Swiss group also supports a positive effect of GM-CSF on CD4+ lymphocytes count [18]. In that trial, the CD4+ T cell counts fell from 720 × 10^6 ^cells/L at baseline to 537 × 10^6 ^cells/L at four weeks after stopping HAART in the STI-only group (p < 0.001). In the GM-CSF treated group, there was no significant change in the CD4+ T cell counts four weeks after stopping HAART. CD4+ T cell counts were 890 × 10^6 ^cells/L at baseline and 792 × 10^6 ^cells/L at week four (p = 0.6). This adds evidence that GM-CSF could have a beneficial effect on CD4+ T cell counts.

#### Impact of GM-CSF on clinical outcomes

GM-CSF has an excellent safety and tolerability profile when used in HIV-1 infected individuals [12,15-17]. In all the major randomized controlled trial, pain, local swelling and erythema were the most frequent side effects and reactions were almost all grade 1 or 2 with only rare grade 3 or 4 events. In the recent randomized controlled trial by Jacobson *et al*, a total of 4 patients had to discontinue GM-CSF use because of toxicity or acute allergic reactions [17]. That was not the case in other trials, where there were no discontinuations of therapy over many patient-months of therapy [12,15,16]. There were no hospitalizations or death attributable to GM-CSF in any study.

The large phase III trial by Angel *et al. *has been the only study to use clinical events as endpoints, using the Centers for Diseases Control and Prevention definition of opportunistic infections (OI), bacterial pneumonia or death as their primary endpoint [12]. An effect of GM-CSF on the rate of OI was not observed, with an event rate of 18% in the placebo group and 21% in the GM-CSF group (p = 0.61). Despite this, there were some important benefits to the use of GM-CSF on other clinical events. These same authors found that the incidence of overall infections (OI and non-OI) was significantly lower in the treatment group of their study; 78% in the placebo group versus 67% in the GM-CSF group (p = 0.03). They also found that time to occurrence of the first infection or death was also significantly longer when GM-CSF was used as an adjunctive treatment in HIV infection (97 days vs. 56 days for placebo; p = 0.04).

For individuals who do not have a history of OI, GM-CSF may decrease the risk of a first opportunistic infection[16]. Despite the fact that they did not observe differences in the rate of overall infections or OI, Brites *et al. *did noticed that all 17 subjects in the GM-CSF arm who developed an OI had a prior history of one or more of these infections. In the placebo group, only 50% of the 14 subjects who developed an OI during the study had a prior history of OI (p < 0.01). This might prove to be an important role for adjunct treatment with GM-CSF as OI are still an important cause of morbidity and mortality in HIV infected individuals.

The most recent randomized control trail by Jacobson *et al. *did show a non-significant reduction in clinical events in the GM-CSF group [17]. No HIV associated clinical events were seen in the treatment group versus 4 in the placebo group (p = 0.12), Again, all the subjects in this trial were on stable HAART prior to and during the study, which is likely responsible for a very low incidence of both overall and OI rates. This, combined with a smaller sample size, likely accounts for the lack of power of this trial to demonstrate an effect of GM-CSF on clinical events.

Finally, the study by Fagard *et al. *failed to show any impact of GM-CSF on clinical events during HAART interruption. However, they studied only 33 patients with high CD4 counts and off HAART for a limited period of time [18].

Despite all these results, questions still remain as to whether use of GM-CSF is associated with a reduction in the incidence of AIDS related morbidity and mortality, as even the authors of the largest phase III study published to date admit to a lack of power in their trial [12]. The introduction of HAART at the time of this trial, thereby likely lowering the incidence of OI in both the GM-CSF group and placebo group, could be expected to have had a significant impact on the outcome of that study.

## Conclusion

In various studies GM-CSF has a positive effect on important parameters of HIV infection, namely plasma HIV RNA levels and CD4+ lymphocytes counts. Although the positive effects are modest and not universally observed, they are significant in many trials. Moreover, the positive effect on these measures may translate into significant clinical benefits. Clinical outcome results of current randomized controlled trials are, thus far, somewhat encouraging. Despite the frequent lack of statistical significance, there are positive trends towards clinical benefit of GM-CSF use in these studies. Moreover, the largest randomized control trial did show that GM-CSF produces a significant reduction in the time to first infection or death. The possibility of allowing longer disease free periods is a desirable outcome for HIV infected individuals, contributing to improved quality of life. However, the high cost of GM-CSF and its mode of administration may be difficult hurdles for patients to overcome.

Future trials designed to look at specific clinical outcomes, for example diseases free period, progression of HIV infection and quality of life, might bring to light additional beneficial effects of GM-CSF. This would require focusing on patients with advanced HIV disease and lower CD4 counts. Alternatively, future trials could focus on the use of GM-CSF as an adjuvant therapy, either to HAART or as an adjuvant with HIV or other vaccines. There is growing evidence that GM-CSF enhances the immune response to vaccines directed against both infectious agents and various cancers [33]. Clinical trials of GM-CSF as an adjuvant to hepatitis B vaccination have shown some positive results [34-37]. Moreover, GM-CSF when added as an adjuvant to HIV envelope vaccination in mice resulted in a greater HIV-specific cellular immune response [38]. Regardless of future studies, it would appear important that those trials focus on individuals with suppressed viral replication, as they seem more likely to realize the benefits of GM-CSF.

It remains to be seen if GM-CSF will ever loose its experimental status and become an accepted therapy for selected individuals HIV infection. The evidence for the role of immunotherapy in HIV/AIDS is ever increasing and GM-CSF might very well become a widely accepted treatment in the years to come.

## Competing interests

The author(s) declare that they have no competing interests.

## Supplementary Material

Additional File 1 Table 1 is a summary of clinical trials of GM-CSF in the treatment of HIV infection. Click here for file
