# Supplementary material for: Granulocyte-macrophage colony-stimulating factor as an immune-based therapy in HIV infection
Source: J Immune Based Ther Vaccines. 2005 May 18;3:3. doi: 10.1186/1476-8518-3-3 (PMC1164429; doi:10.1186/1476-8518-3-3)
Supplement: Additional File 1 — Table 1 is a summary of clinical trials of GM-CSF in the treatment of HIV infection. [file 1476-8518-3-3-S1.doc]

## Table 1. Summary of Clinical Trials of GM-CSF in the Treatment of HIV Infection

| *Study* | *Study Design and Intervention* | *Patient Population* | *Effect on Plasma HIV RNA* | *Effect on CD4 cell count* | *Clinical Outcomes* |
| --- | --- | --- | --- | --- | --- |
| Skowron *et al*.  *J. Infect Dis*, 1999. [15] | Randomized, double-blinded, placebo controlled trial.  Patients on stable, single agent antiretroviral therapy  GM-CSF 250μg thrice weekly vs. placebo for 12 weeks | N=20  Baseline viral load 4.21 log_10_ copies/ml GM-CSF group; 3.95 log_10_ copies/ml in placebo group.  Baseline CD4 count 178 cells/mm^3^ GM-CSF group; 243 cells/mm^3^ placebo. | No significant overall change in mean viral load between groups.  More subjects (50% vs. 10%) in GM-CSF group had a viral load decrease of more than 0.5 log_10_ copies/ml. | Higher in GM-CSF group although no statistical difference.  70% of patients in GM-CSF group (vs. 30% in placebo group) had a CD4 count increase >30% at any given time (p=0.07). | No HIV associated clinical events reported |
| Angel *et al.*  *AIDS,* 2000*.* [12] | Randomized, double-blinded, placebo controlled trial.  All patients had to have received 28 days of stable anti-retroviral therapy; 77% on at least 3 agents.  GM-CSF 250μg thrice weekly vs. placebo for 24 weeks | N=309  Baseline viral load 4.94 log_10_ copies/ml GM-CSF group; 4.98 log_10_ copies/ml in placebo group.    Baseline CD4 count 50.8 cells/mm^3^ GM-CSF group; 49.8 cells/mm^3^ placebo. | No significant overall change in mean viral load between groups.  GM-CSF use was associated with a greater likelihood of maintenance of virologic suppression at 6 months for subjects with <400 copies/ml at baseline. (83% GM-CSF vs. 54% placebo; p=0.02) | Statistically significant increase in GM-CSF group vs. placebo group at 1, 3 and 6 months.  CD4 count of 152 cells/mm^3^ in GM-CSF group vs. 102 cells/mm^3^ in placebo group at 12 months. | No significant difference is AIDS defining events.  Overall infection rate lower in GM-CSF group (67 % vs. 78%; p=0.03).  Time to first infection or death longer in treatment group (97 days vs. 56 days; p=0.04). |
| Brites *et al.*  *J. Infect Dis*, 2000. [16] | Randomized, double-blinded, placebo controlled trial.  All patients were required to have had previous AIDS defining illness within the last three months.  All the subjects were required to be at least on AZT and 66% of subjects were on a 2^nd^ agent.  GM-CSF 125μg twice weekly vs. placebo for 24 weeks | N=105  Baseline median viral load 5.19 log_10_ copies/ml GM-CSF group; 4.97 log_10_ copies/ml in placebo group.  Baseline median CD4 count 80 cells/mm^3^ GM-CSF group; 139 cells/mm^3^ placebo. | Mean plasma HIV RNA change -0.60 log_10_ copies/ml in GM-CSF group vs. -0.07 log_10_ copies/ml in placebo group at week 24 (p=0.02). | Increase of 35 cells/mm^3^ in GM-CSF group vs. 12 cells/mm^3^ in placebo group at week 24 (p=0.42).  80% of subjects in GM-CSF group (vs. 59% in the placebo group) had CD4 count increase of >30% (p=0.03) | GM-CSF use associated with decreased risk of developing a first opportunistic infection (OI); in GM-CSF group all that developed an OI (n=17) had a prior history of OI whereas only 50% in placebo group (n=7/14 with OI) had prior OI (p=<0.01). |

| Jacobson *et al.*  *J Infect Dis.,* 2003 [17] | Randomized, double-blinded, placebo controlled trial.  Stable HAART for minimum of 8 weeks prior to the study and continuing during the study.  GM-CSF 250μg thrice weekly vs. placebo for 16 weeks, followed by open label period of GM-CSF 250μg thrice weekly for another 16 weeks. | N=116  Baseline median viral load 4.07 log_10_ copies/ml GM-CSF group; 4.23 log_10_ copies/ml in placebo  Baseline median CD4 counts 85 and 331 cells/mm^3^ for GM-CSF group; 103 and 353 cells/mm^3^ for placebo group in <200 and ≥200 CD4 cells/mm^3^ strata respectively. | No statistically significant change in viral load, in either CD4 cell counts strata (< or ≥200 cells/mm3). In the combined strata, the change in HIV RNA was +0.048 log_10_ copies/ml in the GM-CSF group vs. –0.103 log_10_ copies/ml in the placebo group at week 16 (p=0.036). | Modest, non statistically significant, increase in CD4+ cell counts in both GM-CSF treated groups vs. placebo. | No HIV associated clinical events in treatment group vs. 4 in placebo (p=0.12). |
| --- | --- | --- | --- | --- | --- |
| Fagard *et al.*  *AIDS,* 2003 [18] | Randomized, controlled trial.  All subjects on stable HAART with viral loads < 50 copies/ml for >6 months and CD4 counts >400 cells/mm^3^ in the month preceding randomization.  GM-CSF 300μg thrice weekly or no intervention for the first 4 weeks during a 12 week interruption of HAART. | N=33  All subjects had viral load < 50 copies/ml at study entry  Baseline median CD4 count 890 cells/mm^3^ GM-CSF group; 720 cells/mm^3^ in control group. | Maximal viral load following HAART interruption lower in the GM-CSF group, 4.97 log_10_ copies/ml compared to 5.54 log_10_ in the control group (p=0.03). | In GM-CSF group, no significant change in CD4 counts despite stopping HAART (890 cells/mm^3^ at baseline and 792 cells/mm^3^at 4 weeks; p=0.6)  In control group, significant decline in CD4 counts during HAART interruption (720 cells/mm^3^ at baseline to 537 cells/mm^3^ at week four; p<0.01) | No HIV associated clinical events reported. |
